# The interaction between the nervous system and the stomatognathic system: from development to diseases

**DOI:** 10.1038/s41368-023-00241-4

**Published:** 2023-08-15

**Authors:** Yuzhu Wu, Yanhua Lan, Jiajie Mao, Jiahui Shen, Ting Kang, Zhijian Xie

**Affiliations:** Stomatology Hospital, School of Stomatology, Zhejiang University School of Medicine, Zhejiang Provincial Clinical Research Center for Oral Diseases, Key Laboratory of Oral Biomedical Research of Zhejiang Province, Cancer Center of Zhejiang University, Engineering Research Center of Oral Biomaterials and Devices of Zhejiang Province, Hangzhou, China

**Keywords:** Extracellular signalling molecules, Bone remodelling, Bone quality and biomechanics

## Abstract

The crosstalk between the nerve and stomatognathic systems plays a more important role in organismal health than previously appreciated with the presence of emerging concept of the “brain-oral axis”. A deeper understanding of the intricate interaction between the nervous system and the stomatognathic system is warranted, considering their significant developmental homology and anatomical proximity, and the more complex innervation of the jawbone compared to other skeletons. In this review, we provide an in-depth look at studies concerning neurodevelopment, craniofacial development, and congenital anomalies that occur when the two systems develop abnormally. It summarizes the cross-regulation between nerves and jawbones and the effects of various states of the jawbone on intrabony nerve distribution. Diseases closely related to both the nervous system and the stomatognathic system are divided into craniofacial diseases caused by neurological illnesses, and neurological diseases caused by an aberrant stomatognathic system. The two-way relationships between common diseases, such as periodontitis and neurodegenerative disorders, and depression and oral diseases were also discussed. This review provides valuable insights into novel strategies for neuro-skeletal tissue engineering and early prevention and treatment of orofacial and neurological diseases.

## Introduction

With the advancement of brain science in recent years, the association between the nervous system and the stomatognathic system has become increasingly evident. To this effect, new concepts, such as neuromuscular dentistry^1,2^ and stomatopsychology^3^ have been proposed to explain the interaction between the two systems. Additionally, research has highlighted the importance of nerves in craniomaxillofacial development,^4^ as well as the crosstalk between nerves and jawbone,^5^ and the diseases that can arise from them.

Anatomically, the nervous and stomatognathic systems are evidently close in proximity. The nervous system consists of the central nervous system (CNS) and the peripheral nervous system (PNS). The former includes the brain and spinal cord, and the latter comprises cranial nerves (linking with the brain) and spinal nerves (linking with the spinal cord).^6^ The peripheral nerves associated with the oral and maxillofacial development region include the trigeminal nerve, facial nerve, glossopharyngeal nerve, vagus nerve, accessory nerve, hypoglossal nerve and even cervical spinal nerves.^7^ The nervous system regulates the stomatognathic system in a variety of ways, from maxillofacial bones to dental pulp, periodontal ligament (PDL), muscles, glands, oral mucosa, the tongue, the temporomandibular joint (TMJ), mouth, skin, and other structures.^8^ This intricate regulation of the nervous system is vital for the proper development and functioning of the maxillofacial system. Maxillofacial deformity and skeletal dysplasia are common comorbidities in neurodevelopmental deficit patients, such as trisomy 21 (ref. ^9^), neurofibromatosis,^10^ and achondroplasia.^11^

The regulation between nerves and bones has been widely studied,^12^ with intrabony nerves being found in cortical bone,^8^ trabecular bone, periosteum, and bone marrow.^13,14^ The CNS regulates bone metabolism through the peripheral autonomic nervous system (ANS) and sensory nerves. The ANS comprises the sympathetic nervous system (SNS) and the parasympathetic nervous system (PSNS).^15,16^ All peripheral nerves regulate bone development and recover via neurotransmitters, neuropeptides, neurotrophins, and others.^17^ In the case of the jawbone, nerves not only distribute in the same parts as other bones, but also in special parts, such as the subchondral condyle, PDL, and dental pulp.^18^ In addition to classic targets, such as osteoclasts and osteoblasts, these parts are also targets of the nervous system that mediates jawbone remodeling. The regulation of nerves on the oral and maxillofacial systems is unique and significant due to the presence of more targets. Furthermore, because of the special anatomy of the jawbone—branches of the trigeminal nerve travel in the intraosseous canals and innervate peripheral tissues,^19^ concomitant peripheral nerve injury can be caused by jawbone defects, and bone repair is accompanied by nerve repair.^20^

The proximity of anatomical structures, and the rich circulatory system of the brain and maxillofacial region, enable the nervous system and the stomatognathic system to interact with each other. The decline or loss of neurological function can result in some oral symptoms, such as facial paralysis^21^ and salivation.^22^ Conversely, oral diseases can influence the nervous system. If oral bacteria intrude into the brain via hematogenous spread, caries, periodontitis, and other oral infections may lead to intracranial infection and even neurodegenerative and neuropsychological diseases.^23^ Oral squamous cell carcinoma (OSCC) and adenoid cystic carcinoma (ACC) can lead to perineural invasion (PNI) of the head and neck as well, resulting in numbness, pain, or dysfunction.^24^ More importantly, the mechanism of some systemic diseases, such as Alzheimer’s disease (AD) and Parkinson’s disease (PD), are too complex to recognize their initiating lesions. Some nervous system diseases and stomatognathic diseases can promote each other, such as depression and periodontal disease,^25^ and pain caused by neuropathy and stomatognathic lesions.^26^ Although abnormalities in the stomatognathic system are not the major cause of neurological diseases, it is important to note that the abnormalities can contribute to their progression. Therefore, understanding the potential links between these two systems is essential for early diagnosis and improved prognosis.

This review provides a comprehensive analysis of the cellular and molecular regulatory mechanisms between nerves and maxillofacial cells during growth and in both physiological and abnormal environments. It further examines the development of the oral and maxillofacial systems, wound healing, and other visible changes from a macro perspective. Additionally, it summarizes the nervous system diseases and disorders caused by the oral and maxillofacial systems, as well as the complex diseases that are strongly linked to the interaction between the nervous and stomatognathic systems. By gaining a better understanding of these complex scenarios, we can further investigate the underlying mechanisms and apply them to clinical settings for the early prevention and treatment of diseases in the future.

## The physiological growth and developmental anomalied of nervous and craniomaxillofacial systems

### Physiological growth of nervous and craniomaxillofacial systems

It has been reported that cranial and maxillofacial development in vertebrates is closely related to neural growth.^4^ During this process, neural crest (NC) cells play a pivotal role, which are characterized by their multi-potential, migration, and differentiation abilities. In early embryonic development, NC cells first appear on the dorsal side of the neural tube and initiate the expression of NC signature genes (FoxD3, Sox10, etc.), signifying the formation of true NC cells.^27,28^ Subsequently, NC cells undergo an epithelial-to-mesenchymal transition to migrate extensively during the entire embryonic development. NC cells can be divided into four main groups along the cephalic and caudal axis: cranial, vagal, trunk, and sacral ganglion subgroups.^29^ Among them, cranial neural crest (CNC) cells, derived from labeling NC cells with Wnt1, are the most significant group involved in craniofacial development, and the only group related to cranial bone formation.^30^ The migration of CNC cells is highly regulated and occurs along well-defined pathways, terminating in the ventral part of the brain and the branchial arch. CNC cells first migrate as continuous waves and rapidly split into three discrete streams to fill the first, second and third branchial arches. Subsequently, CNC cells contribute to various structures, including the skeletal system (cartilage and jawbone), cranial nerves and ganglia, as well as smooth muscle, vascular connective tissue, and the dermis of the head.^31^ Moreover, CNC cells form multiple components of the tooth through sequential and induced epithelial-mesenchymal interactions between odontogenic mesenchymal cells derived from CNC and the covering ectoderm.^32^ Consequently, nerves play a crucial role in cranial and maxillofacial development.

#### Developmental anomalies of the nervous and craniomaxillofacial systems

There are many congenital or genetic diseases that have multiple concurrent developmental alterations affecting the nervous system and stomatognathic system, some definitely serious for survival and others with less dramatic prognoses for life. Here are three of the typical diseases, and Table 1 lists additional ones.

**Table 1:** Congenital diseases with neurologic disorder and cranio-facial abnormalities

Disease	Etiology	Pathogenesis	Nervous system symptoms	Stomatognathic symptoms	References
Trisomy 21	Neurodevelopmental disorders	An extra copy of human chromosome 21, abnormal expression of non‑HSA21 genes and deregulated non-coding genetic elements	Deficits in short-term memory skills, exhibit various language weaknesses	Dental caries, missing teeth, malformed teeth, delayed teeth eruption, malocclusion, periodontitis, fissured lips and tongue, macroglossia, mouth breathing and bruxism	^9,33–41^
NF1	Direct infiltration or downward traction by neurofibromas	NF1 gene mutations → RAS-MAPK pathway ↑ → cell hyperproliferation, tumor predisposition ↑	Neurofibromas, optic pathway gliomas, astrocytomas, and malignant peripheral nerve sheath tumors	Jaw malformations, malocclusion, malformed nose and upper lip, gingival enlargement, gingival neurofibroma, nodular lesions on the tongue, and perineural fibrous thickening within the dental pulp	^10,42–50^
Achondroplasia	Premature synchondrosis closure, and impaired endochondral ossification	FGFR3 gene mutations → Activated FGFR3 signaling → Bmp ligand ↑ → bone formation ↑ synchondrosis closure ↑	Neurologic deficits: myelopathy, radiculopathy, neurogenic claudication	Bilateral or unilateral facial paralysis, prominent forehead, midface hypoplasia, occlusal abnormality, low nose bridge, narrow nasal passages	^11,51–54^
Edwards syndrome (trisomy 18 syndrome)	One of the autosomal trisomy syndromes, an extra copy of chromosome 18q	three copies of two critical regions in the long arm of chromosome 18, 18q12.1 to 18q21.2 → mental retardation	Delayed psychomotor development and mental retardation, epilepsy, cerebellar hypoplasia, meningoencephalocele, anencephaly, holoprosencephaly, hydrocephalus, hypoplasia of the corpus callosum	Microcephaly, bitemporal narrowing, micro-retrognathia, asymmetric face with facial paralysis, microstomia, narrow arched palate, cleft lip, cleft palate.	^230,231^
Noonam syndrome	Disease-causing mutations of eight genes (PTPN11, SOS1, KRAS, NRAS, RAF1, BRAF, SHOC2, and CBL) in the RAS–MAPK pathway	PTPN11 mutations (50% of N00nam syndrome) → protein SHP2 constitutive or prolonged activation → development anomalies.	Intellectual impairment, emotional perception difficulties, language impairment	Prominent nasolabial fold, deeply grooved philtrum, high wide peaks of the vermilion, micrognathia, poor suck	^232–235^
Williams syndrome	Mispairing of low-copy DNA repetitive elements at meiosis	Base pair microdeletion on chromosome 7q11.23 → affect gene transcription and DNA methylation → glycolysis and neuronal migration-associated gene dysregulation	Intellectual disability, motor deficits, hypersociability, memory decline	Broad forehead, flat nasal bridge, long philtrum, micrognathia	^236,237^
Prader-Willi syndrome	Hydrocarbons → chromosomal damage, deletion of imprinted genomic	Errors of genomic imprinting: lack of expression of paternally inherited imprinted genes in the chromosome 15q11-q13 region, maternal uniparental disomy 15	Mood disorders, cognitive impairment, psychosis, autistic spectrum disorder, intellectual delay, epilepsy, stunting, behavioral problems, hypothalamic dysfunction	Craniofacial deformities, narrow nasal bridge, thin upper lip vermillion, down-turned corners of the mouth, dry mouth, dry mucosal membranes, sticky salvia, poor suck, enamel hypoplasia, dental caries, bruxism.	^238,239^
Crouzon syndrome	Craniosynostosis	FGFR-2 and FGFR-3 gene mutations → protein function ↑ → osteoblast differentiation ↑ → bone formation ↑ → craniosynostosis	Hydrocephalus, optic atrophy, papilledema	Brachycephaly, orbital hypoplasia, maxillary hypoplasia, high arched palate, dental dimensions↓	^86,240^
Apert syndrome	Craniosynostosis	mutation in amino acid residues (p.Ser252Trp or p.Pro253Arg- linker region bridging Ig-like domains II and III of FGFR2) → FGFR2 activation ↑ → cell proliferation, differentiation ↑ → osteogenesis ↑	Mental retardation, ventriculomegaly, Abnormalities of midline development, malformations of cortical development, white matter bulk ↓ , temporal lobe abnormalities, encephalocele	mid-facial hypoplasia, maxillary hypoplasia more severe (compared to Crouzon syndrome), crowded maxillary dentition, mandibular rotate clockwise, anterior open bite, and congenitally missing teeth, high arched palate, palatal swelling, soft cleft palate	^241–243^
Kabuki syndrome	Functional neurological abnormalities → muscular hypotonia → oral motor dysfunction	KMT2D and KDM6A gene mutations → abnormal histone expression → abnormal transcriptional regulation	Intellectual disability, cognitive impairment, epilepsy, hearing loss neuroblastoma, spinal ependymoma	Oral motor dysfunction (dysarthria, poor coordination in sucking and swallowing), arched eyebrows, long palpebral fissures, a broad or depressed nasal tip with hypoplastic columella, cleft palate, cleft lip, congenital absence of teeth	^244–246^
Moebius syndrome	Hypoplasia and atrophy of cranial nerve nucleus	Primary genetic and ischemic cause	Paralysis of the abducens and facial cranial nerves	Bilateral or unilateral facial paralysis, microstomia, hypotonic lip muscles, tongue deformity, dysfunction of palate and pharynx, dental enamel hypoplasia, open bite or deep overbite, high arched palate, and maxillary and mandibular hyperplasia.	^247–250^
Parry–Romberg syndrome	Autoimmune disease	Abnormal developmental migration of NC cells, trigeminal peripheral neuritis, neurotrophic viral infection and other dysfunctions of the peripheral SNS	Cognitive impairment, behavioral disorders, seizures, intracranial vascular malformations, aneurysms, brain atrophy, cranial neuropathies, hemiplegia, migraines, and trigeminal neuralgia	Gingiva, tongue, and soft palate involvement, dental root exposure or resorption, delayed tooth eruption, mandibular atrophy and abnormal TMJ.	^251,252,253^
Anderson syndrome	Ion channel defect	KCNJ2 mutations → Kir2.1 ion channels ↓ → the terminal phase of action potential ↑ → L-type Ca2+ channels ↑ → arrhythmias↑ Not clear: Kir2.1 channels ↓ → skeletal abnormalities	Periodic paralysis	Hypertelorism, small mandible, cleft palate.	^254–256^

*NF1* neurofibromatosis type 1, *RAS* rat sarcoma, *MAPK* mitogen-activated protein kinase, *FGFR* fibroblast growth factor receptor, *FGF* fibroblast growth factor, *KMT2D* histone-lysine N-methyl-transferase 2D, *KDM6A* lysine (K)-specific demethylase 6A, *NC* neural crest, *SNS* sympathetic nervous system, *TMJ* temporomandibular joint

##### Trisomy 21

Trisomy 21 (Down syndrome) is a genetic disorder resulting from an extra copy of human chromosome 21, occurring at a frequency of 1:600 to 1:2 000 (ref. ^33^). In fact, abnormal expression of non‑HSA21 genes and deregulated non-coding genetic elements also influences brain and cognitive development in Trisomy 21. Patients with Trisomy 21 often suffer from mental retardation, neurodevelopmental disorders, and even AD with age.^9^ They typically exhibit deficits in short-term memory and language abilities, as well as a variety of oral symptoms such as periodontitis, angular lip cheilitis,^34^ missing teeth, malformed teeth, delayed tooth eruption, malocclusion, fissured lips and tongue, macroglossia, mouth breathing, and bruxism.^35^ The etiology of hypodontia abnormal development of the teeth may refer to alterations in the PNS^36^ or the abnormalities in tooth germs.^37^ Inflammation, on the other hand, can be linked to alterations in patients’ immune response^38^ or various systemic or infectious diseases.^39^ Although novel treatments are being investigated, treatment of Trisomy 21 is largely based on approaches used for other diseases, such as AD.^40^ And craniofacial or dentoalveolar aesthetics of patients with Trisomy 21 can be improved with surgical procedures and orthodontic treatments.^35,41^

##### Neurofibromatosis type 1

Many reports have demonstrated concomitant morpho-functional alteration in the stomatognathic system in individuals with neurofibromatosis. Neurofibromatosis is divided into two types: type 1 and type 2, the more common being Neurofibromatosis type 1 (NF1), which occurs at a frequency of 1 in 1000. NF1 is an autosomal dominant inherited disorder, and its pathogenesis is associated with mutations of the NF1 gene, which encodes the tumor suppressor neurofibromin.^42,43^ These mutations lead to the hyperactivation of the rat sarcoma mitogen-activated protein kinase (RAS-MAPK) pathway, which provokes cell hyperproliferation or tumorigenesis, like neurofibromas, optic pathway gliomas, astrocytomas, and malignant peripheral nerve sheath tumors.^10^ Because NF1 affects the underlying facial skeleton and can even directly infiltrate or pull down surrounding tissues, midface deformity is common in NF1 patients.^44^ Oral manifestations can be found in approximately 72% of NF1 patients,^45^ with hard tissue (jawbone and teeth) malformations like intrabony cystic lesions, enlarged or branched mandibular canals^46^ and malocclusion remaining prominent across the board.^47^ In addition, soft tissue deformities are frequently seen due to the morphological variations in particular sites. Examples of such deformities include malformed nose and upper lip areas, gingival enlargement,^48^ gingival neurofibroma,^45^ nodular lesions on the tongue,^49^ and perineural fibrous thickening within the dental pulp.^50^ Due to a broad spectrum of lesions associated with NF1, surgical resection is usually used for therapy^44^

##### Achondroplasia

The formation of mammalian skeletons occurs via intramembranous or endochondral ossification. The former occurs in the midface and the latter occurs in the skull base and nasal septum.^51^ Achondroplasia is the most prevalent genetic disorder of dwarfism, occurring at a frequency of 1 in 26,000 (ref. ^52^). Its pathogenesis is linked to activating mutations in the gene encoding fibroblast growth factor receptor 3 (FGFR3),^53^ which is a pivotal regulator of endochondral bone growth. Activated FGFR3 signaling in chondrocytes increases the expression of Bmp ligand mRNA, which promotes osteoblast differentiation and accelerates bone formation and synchondrosis closure. Furthermore, the early closure of synchondroses may lead to the narrowing of the foramen magnum and spinal canals,^54^ resulting in severe neurological complications, including radiculopathy, myelopathy, and neurogenic claudication. In terms of maxillofacial symptoms, achondroplasia patients may have a prominent forehead, midface hypoplasia, occlusal abnormality, low nose bridge, narrow nasal passages, all of which are caused by defective endochondral ossification in craniofacial cartilage and premature closure of the growth center in craniomaxillofacial skeletogenesis.^11^ Due to critical illness in the nervous and orofacial system, any intervention ought to be implemented before the synchondrosis closure.

## Homestasis and regulation between the nervous system and jawbones

### Effect of nerves on jawbones

**Fig. 1:**
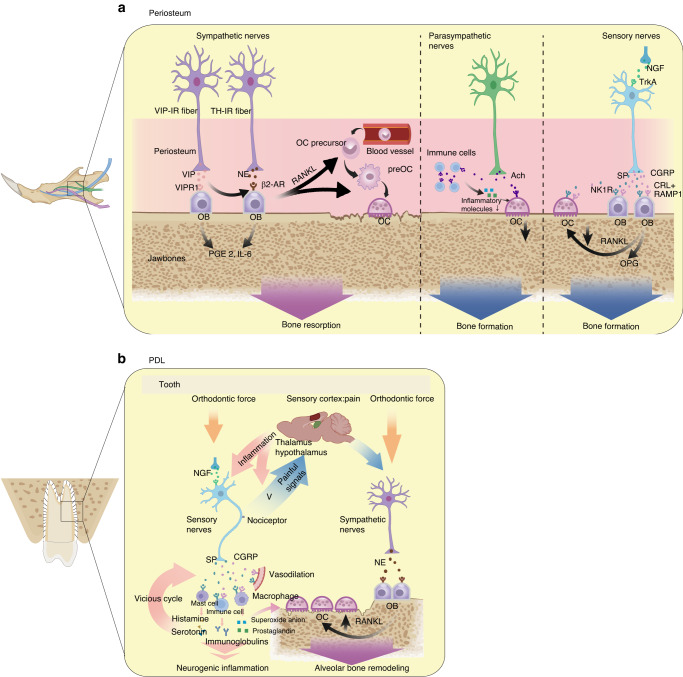
Effect of nerves on jawbones. **a** Effect of ANS and sensory nerves on jawbones. VIP and NE released from sympathetic nerves can activate corresponding receptors and upregulate RANKL in OBs, and RANKL contributes to OC maturation. All of them lead to bone resorption. Ach released from parasympathetic nerves may contribute to anti-inflammatory activity and osteoclast apoptosis. CGRP and SP released from sensory nerves downregulate RANKL and upregulate OPG in OBs, thereby hastening bone formation. **b** The neurofeedback in the PDL under the induction of orthodontic force. Orthodontic force triggers nociceptors in sensory fibers, leading to inflammatory cascade mediated by CGRP and SP, as well as the activated neural loop of the sensory-central-SNS. Orthodontic force also activates sympathetic nerves and promotes osteoclast activity. The neurofeedback influences alveolar bone remodeling and tooth movement. PGE 2 prostaglandin E2, IL-6 interleukin-6, Ach acetylcholine, NK1R neurokinin 1 receptor, CRL+RAMP1 calcitonin receptor-like receptor+receptor activity-modifying protein 1, V trigeminal nerve

The anatomical structure of the jawbone is unique: the nerves travel in the bony ducts and send branches directly to surrounding tissues. The trigeminal nerve, the largest cranial nerve, comprises the ophthalmic, maxillary, and mandibular branches.^55^ The maxillary nerve innervates the maxilla, and the inferior alveolar nerve (IAN), which is the largest branch of the mandibular nerve, innervates the mandible.^56^ In addition to branches of the trigeminal nerve, ANS also plays a significant role in the physiology and pathology of the jawbone.^57^ Experimental animal studies have shown that the complex and intricate mechanism involves various nerves and bioactive factors secreted within the microenvironment.^57,58^ In particular, intrabony nerves regulate jawbone metabolism through neurotransmitters, neuropeptides, neurotrophins, and other signaling molecules.^59,60^ The tyrosine-hydroxylase-immunoreactive (TH-IR) fibers and vasoactive intestinal polypeptide (VIP)-IR fibers are sympathetic fibers. The TH-IR and VIP-IR fibers secrete norepinephrine (NE) and VIP respectively. Sensory neurons secrete calcitonin gene-related peptide (CGRP) and substance P (SP), so CGRP-IR fibers and SP-IR fibers are sensory fibers.^61^ The accumulation of various biological factors within the microenvironment of jawbones, along with the presence of their receptors in osteogenic and osteoblast lineage cells,^62–64^ provides compelling evidence of bilateral homeostasis between nerves and the jawbone (Table 2 and Fig. 1).

**Table 2:** Published studies on the effect of nerves on jawbones

Types of nerves		Fibers/Neurotransmitter	Signaling pathways	Function	References
ANS	SNS	Catecholaminergic innervation (TH-IR fibers)/NE	Distributed within the mandibular endosteal retromolar zone. NE → osteoblast with β2-ARs activation → RANKL and pro-resorbing factors ↑ → osteoclast differentiation ↑	Osteoclasis	^5,57,62,65–69,257,258^
		Cholinergic innervation (VIP-IR fibers)/VIP	Distributed within mandible periosteum and alveolar wall. VIP → osteoblast with VIPR 1 activation → prostaglandin E-2 ↑ , the activating effect of the pro-resorbing factors ↑	Osteoclasis	^5,61,259,260^
	PSNS	Ach	PSNS activation → OC ↓ → bone loss ↓ Baroreflex and chemoreflex ↑ → PSNS activation ↑ → anti-inflammatory → OC ↓ → bone loss ↓	Osteogenesis	^66,72–78^
Sensory nerves		CGRP	Dilates blood vessels and induces angiogenesis, CGRP → osteoblasts and progenitor cells with receptors → osteoblasts survival ↑ OPG/RANKL ratio ↑ → osteoclast ↓	Osteogenesis	^79,261^
		SP	SP → BMSCs with NK1-R → proliferation and osteoblastic differentiation ↑ →; osteogenesis SP → osteoclasts with NK1-R → osteoclast activity ↑	Osteogenesis and osteoclasis	^64,81,82,80^
		NGF	Axons regeneration ↑ → bone formation ↑ NGF → osteoblasts differentiation↑	Osteogenesis	^83–85^

*ANS* autonomic nervous system, *SNS* sympathetic nerve system, *PSNS* parasympathetic nervous system, *TH-IR* tyrosine-hydroxylase immunoreactive, *NE* norepinephrine, *RANKL* receptor activator for nuclear factor-KB ligand, *β2-ARs* beta-2 adrenergic receptors, *VIP-IR* vasoactive intestinal peptide immunoreactive, *VIPR 1* VIP receptor 1, *Ach* acetylcholine, *CGRP* calcitonin gene-related peptide, *OPG* osteoprotegerin, *SP* substance P, *NK1-R* neurokinin 1 receptor, *NGF* nerve growth factor

#### Autonomic nervous system

Animal experiments show that SNS negatively affects bone mass,^65^ whereas PSNS does the opposite.^66^ Previous research indicates that heightened SNS activity causes bone loss.^67^ SNS promotes bone resorption through the released NE and active β2-adrenergic receptors (β2-ARs),^62^ as well as the receptor activator of nuclear factor kappa B ligand (RANKL)—osteoprotegerin (OPG) system.^68^ The impact of SNS on the jawbone is more complicated than previously reported. Both TH-IR fibers and VIP-IR fibers distribute within the mandible periosteum and alveolar wall, but the distribution of TH-IR fibers is wider, and includes the mandibular endosteal retromolar zone. NE and VIP are two bioactive factors that contribute to osteoclast differentiation and bone resorption. Following sympathectomy, the number of TH-IR fibers and VIP-IR fibers declines, while the number of CGRP-IR fibers increases,^61^ which is associated with sensory-sympathetic interactions mediated by neurotrophic factors.^69^ Sympathectomy changes the expression of NGF and semaphorin 3A (sema3a), leading to the increase of CGRP-IR fibers.^70^ Following a superior cervical ganglionectomy in female rats, bone mineral density increased significantly.^57^ This can be attributed to the inhibition of the SNS, which decreases the number of RANKL-expressing osteoblasts and preosteoclasts in the mandibular periosteum, thereby facilitating osteogenesis.^5^ Nerve fibers also innervate the TMJ, and active sympathetic signaling has been found to be related to bone loss during osteoarthritis of the TMJ, whereas the use of β2-ARs antagonists can suppress subchondral bone resorption and osteoclast function.^71^ Therefore, the metabolism of different regions of the jawbone is modulated by the sympathetic pathways.

In addition, the relationship between ANS and immune response has been investigated in the alveolar bone.^72^ Acetylcholine (a neurotransmitter secreted by PSNS) and its receptors have been found to be expressed in various non-neuronal cells including human keratinocytes,^73^ fibroblasts, T cells, B cells and macrophages.^74,75^ Clinical data and animal experiments reveals that acetylcholine can regulate inflammation-related cells by activating the α7 nicotinic receptor, which promotes anti-inflammatory activity^75^ and reduces the release of inflammatory factors.^76–78^ In fact, PSNS activation can promote osteoclast apoptosis to favor bone mass accrual.^66^ It has been found that electrical activation of the carotid sinus nerve can alleviate alveolar bone loss and periodontal disease in rats. This effect may be attributed to activation of PSNS and its anti-inflammatory response by provoking baroreflex and chemoreflex.^72^ However, comprehensive and thorough research investigating the regulation of ANS on the jawbone is relatively scarce. Therefore, further exploration is needed to understand the effect of ANS on the jawbone and its underlying mechanism.

#### Sensory nerves

The role of sensory nerves should not be ignored in bone regeneration. At the micro‐level, these nerves promote bone recovery through the release of neuropeptides, such as CGRP and SP. Their receptors are expressed on bone cells,^5,63,64^ indicating a strong association between the nervous system and bone metabolism in animal models. CGRP is a positive mediator for bone modeling, as it suppresses the number of osteoclasts by regulating the OPG/RANKL ratio. CGRP also promotes the osteogenic differentiation of human PDL stem cells to repair rat alveolar bone defects.^79^ However, the effect of SP appears to be contradictory. In vitro, studies indicate that SP can stimulate osteoblast and osteoclast differentiation and function.^80^ In vivo, studies show that a combination of SP and calcium phosphate cement can contribute to alveolar bone defect restoration.^81^ Additionally, SP has been found to hasten bone formation during mandibular distraction osteogenesis.^82^ Nonetheless, SP can inhibit osteogenesis induced by lipopolysaccharide from Porphyromonas gingivalis.^64^ Generally, CGRP and SP act synergistically since they are frequently co-localized in the same fibers and bone defect sites and released synergistically. After transection of the IAN, the secretion of CGRP and SP decreases,^58,59^ which reduces the OPG/RANKL ratio and promotes osteoclastogenesis. Thus, injured or transected IAN result in sensory nerve degradation and mandibular bone destruction. Nerve growth factor (NGF), a key neurotrophin released by sympathetic and sensory nerves,^83,84^ has been found to stimulate bone formation by inducing regenerating axons,^85^ and consequently, improving the density and quality of new bone in a rabbit model of mandibular distraction osteogenesis.^86^ Altogether, these findings indicate that sensory nerves play a significant role in bone formation and regeneration (Fig. 1a).

In addition to the classical pathways of neural regulation, such as those of limb bones, jawbone remodeling is also regulated by neural signals within the PDL.^87^ The PDL is the soft tissue between the teeth and alveolar bone, and it serves as a critical anatomical structure in orthodontic treatment. It has been reported that fibroblasts and osteoblasts in the PDL may respond directly to mechanical forces and initiate the remodeling of alveolar bone^88,89^ through mechanotransduction^90,91^ and intracellular signaling cascades.^92,93^ Additionally, the PDL is abundantly supplied with sympathetic, parasympathetic and sensory fibers,^94,95^ which contribute to alveolar bone remodeling and tooth movement. As mentioned before, sympathetic fibers release NE and VIP to promote bone resorption, while parasympathetic fibers secret acetylcholine to inhibit bone resorption.^66^ Thinly myelinated and unmyelinated sensory fibers express CGRP and SP to facilitate osteogenesis.^87^ Sensory fibers in the PDL contain nociceptors,^96^ which are triggered by orthodontic force, resulting in transmission of painful signals to the brain.^97–99^ This process activates an inflammatory cascade in the trigeminal spinal nucleus.^87^ It is mediated by the activation of neurons and inflammatory cells,^100,101^ leading to an increase in the secretion of NGF,^102^ CGRP,^103^ SP^104^ and various inflammatory molecules.^87^ In addition, the activated neural loop of the sensory-central-SNS influences orthodontic tooth movement.^105^ In summary, the PDL is a complex system, and nerves within it play a critical role in tooth movement and alveolar bone remodeling (Fig. 1b).

### Regulation of jawbones to nerves

The condition of the jawbone can also affect the distribution of nerves.

#### Anatomical factors

The presence of teeth and the intraosseous canal makes the jawbone unique compared to other bones, and also affects nerve distribution. The mandibular canal is a compact bone canal in the cancellous bone of the mandible. The IAN runs through the mandibular canal and sends branches to control the teeth in what are known as mandibular canal branches. The number of these mandibular canal branches is largely determined by the number of teeth and occlusion elements in the human mandible.^106^ Since the presence of teeth helps to maintain the alveolar bone matrix,^107^ when teeth are lost, nerve branches may disappear due to the absorption of alveolar bone.^106,108^

#### Mechanical factors

Actually, nerves can sense and respond to mechanical signals, which include the rigidity of the environment and press/traction exerted on the neurons by neighboring cells.^109^ The latter signal includes the tension of the jawbone and the orthodontic force of the teeth. After mandibular distraction osteogenesis, the elongation of the IAN occurs along with mandible regeneration in dogs.^110^ Aside from traction on the mandible, the orthodontic force on the teeth can also affect the distribution of nerves in the PDL, which is a specialized fibrous connective tissue, and dental pulp, which is connected to the PDL through the dentinal tubules and apical foramen. Dental pulp and PDL are richly supplied with sensory and sympathetic nerve fibers. They also feature immunoreactivity to protein gene product 9.5 and CGRP.^95,111^ It has been demonstrated that the reaction of the PDL is directly related to the duration, type, direction, and magnitude of the force on the teeth.^112,113^ Appropriate and intermittent orthodontic force will not cause permanent damage for the PDL and pulp.^114^ The density of nerve fibers in the pulp and PDL increases initially and then recovers as the duration of the force increases. However, constant, or excessive force may lead to irreversible damage of the PDL, and even cause pulp necrosis and root resorption.^115^ Injury to the IAN and related neuropathy is rare during orthodontic treatment. However, the roots of molar or premolar teeth are situated in close proximity to the IAN, the IAN may be injured.^116^

#### Bioactive factors

Bioactive signaling factors secreted by bone lineage cells have the potential to modulate the physiological activity of the nerves. Osteoblastic cells express NGF and sema3a. The former is a nerve attractant molecule involved in nerve fiber maintenance and plasticity,^117^ and the latter is a repulsive molecule that inhibits fiber sprouting.^118,119^ The molecular network is disrupted after sympathectomy and the subsequent loss of VIP expression, leading to changes in the expressions of NGF and sema3a in rat mandible. As a result, CGRP-positive fibers invade the osteogenic layer due to the decrease in pro NGF and sema3a, and CGRP-positive fibers increase in the periosteum non-osteogenic layer due to an increase in mature NGF.^70^

## Non-developmental diseases caused by reciprocal regulation between the nervous system and the stomatognathic system

### Craniofacial diseases caused by neurological illnesses

**Fig. 2:**
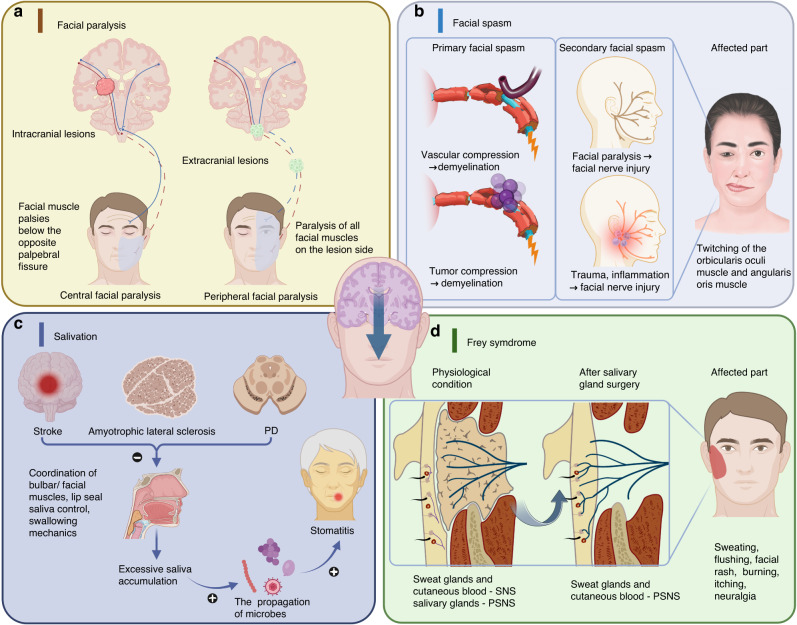
Craniofacial diseases caused by neurological illnesses. **a** Facial paralysis. Lesions located between the cerebral cortex and the facial nerve nucleus lead to central facial paralysis. Extracranial lesions cause peripheral facial paralysis. **b** Facial spasm. Demyelination cause primary facial spasm and facial nerve injury may result in secondary facial spasm. **c** Salivation. Weakness or poor coordination of bulbar or facial muscles resulted from neurological diseases can cause salivation. **d** Frey syndrome. After parotid gland surgery, PSNS fibers can control sweat glands and blood vessels in the skin, leading to sweating and flushing during chewing. Created with BioRender.com

Several main oral symptoms arise from the decline or loss of neurological function, such as facial paralysis, facial spasm, salivation, and Frey syndrome (Fig. 2).

#### Facial paralysis

Facial paralysis is a typical neuro-stomatology disease that is divided into central facial paralysis and peripheral facial paralysis. Facial paralysis is caused by a dysfunction of the facial nerve, leading to the limitation of the activity of the facial muscles innervated by the nerve.^120^ Central facial paralysis lesions are located between the cerebral cortex and the facial nerve nucleus. Common etiologies include cerebrovascular diseases, intracranial tumor compression, brain trauma, and congenital facial nerve dysplasia.^121–123^ Symptoms of central facial paralysis manifest in facial muscle palsies below the opposite palpebral fissure, disappearance of the nasolabial fold, and food retention in the oral vestibule. Peripheral facial paralysis is more commonly caused by extracranial etiologies, including viral infections (especially herpes zoster virus),^124^ parotid malignant tumors, trauma, and even cold wind.^125,126^ Bell palsy is the most prevalent type of peripheral facial paralysis.^127^ Symptoms of Bell palsy include paralysis of all facial muscles on the lesion side, disappearance of forehead lines, inability to close the eyelids, sagging of the mouth angles, and even accompanying auditory changes and hypogeusia (Fig. 2a).^128^

#### Facial spasm

Facial spasm refers to involuntary convulsions or spasms^129^ of half of the facial muscles. It is classified as primary and secondary facial spasm.^130^ Primary facial spasm arises from demyelination caused by cerebellar pontine angle tumors^131^ and vascular malformations that compress the facial nerve root.^132,133^ This demyelination disrupts the normal flow of action currents along the nerve fiber, resulting in overexcitation of the facial nerve and subsequent facial spasm.^134^ Secondary facial spasm is caused by facial nerve injury due to facial paralysis, trauma, inflammation, and other factors.^130^ The twitching typically begins with the orbicularis oculi muscle and gradually extends to other facial expression muscles on the affected side.^135^ And the twitching of the angularis oris muscle is the most prominent symptom (Fig. 2b).^129,136^

#### Salivation

Saliva is secreted by salivary glands, which are stimulated by the PSNS, but the contraction of the salivary duct’s smooth muscle is controlled by the SNS. Therefore, neurological lesions can cause abnormal salivary secretion. The etiology of salivation may refer to weakness or poor coordination of bulbar or facial muscles, leading to poor lip seal, ineffective saliva control, and impaired swallowing mechanics.^137^ Therefore, neurological conditions like stroke, neuromuscular diseases like amyotrophic lateral sclerosis, and neurodegenerative diseases including PD, multiple system atrophy, and cerebral palsy can cause salivation.^22^ Excessive saliva accumulation in the mouth corner leads to a rapid propagation of microbes such as Candida albicans, Streptococcus spp, Staphylococcus spp, and herpesvirus, resulting in oral mucosal diseases, such as candidal stomatitis, coccal stomatitis, and herpes stomatitis (Fig. 2c).^138–140^

#### Frey syndrome

The salivary glands receive signals from the PSNS, while the sweat glands and cutaneous blood vessels are regulated by the SNS.^141^ Physiologically, saliva secretion and sweating are two separate processes. The salivary gland secretes saliva in response to chewing stimulation, while there is no significant change in the skin condition. However, after parotid gland surgery, PSNS fibers can control denervated sweat glands and blood vessels in the skin.^142^ Therefore, chewing can lead to not only saliva secretion from other salivary glands, but also sweating and flushing in the preauricular area due to increased PSNS activity. This phenomenon is known as Frey syndrome,^143^ which is characterized by sweating and flushing in response to mastication or a salivary stimulus.^144^ In fact, it is common symptom following salivary gland surgery.^145^ And other symptoms include face rash,^146^ burning, itching, forehead and scalp sweating^147^ and neuralgia (Fig. 2d).^144^

### Neurological diseases caused by an aberrant stomatognathic system

**Fig. 3:**
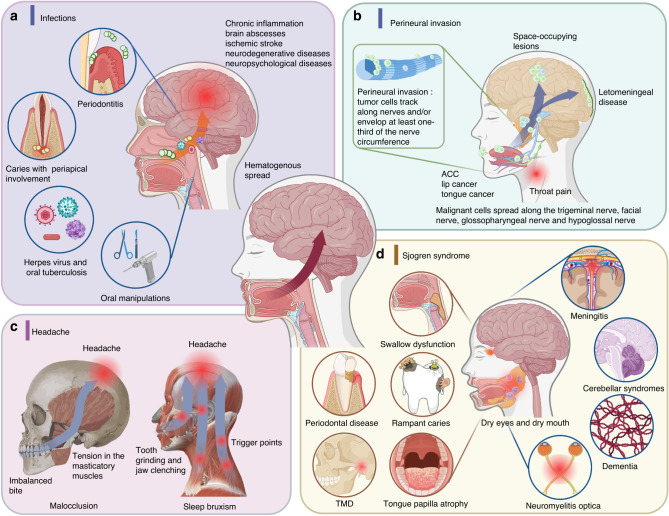
Neurological diseases caused by an aberrant stomatognathic system. **a** The link between oral infection and CNS infection. Oral microbes are easy to invade the brain via hematogenous spread. **b** Perineural invasion resulting from tumors in the oral and maxillofacial regions. **c** Headache caused by malocclusion and sleep bruxism. **d** Sjogren syndrome. Mononuclear cells and lymphocytes invade lacrimal and salivary glands. Created with BioRender.com

While stomatognathic system abnormalities may not be the primary cause of neurological diseases, it is important to consider the potential links between them. Craniofacial symptoms or diseases, such as oral infection, OSCC, malocclusion and Sjogren syndrome (SS), can play a role in the development of neurological diseases. A comprehensive understanding of these links can aid in early prevention and treatment of these neurological diseases (Fig. 3).

#### The link between oral infection and CNS infection

The presence of abundant microflora in the oral cavity,^148^ combined with anatomical proximity of the brain and maxillofacial region, makes the CNS susceptible to infection. In analogy to the “gut-brain axis”, the proposed concept of the brain-oral axis suggests the profound influence of an oral microbiome on the brain.^23,149^ Hematogenous spread is the predominant mode of intracranial dissemination, and caries with periapical involvement and periodontitis are the most frequently-triggering factors.^150^ In addition, other oral and maxillofacial specific infections, including herpes simplex,^151^ herpes zoster, hand-foot-mouth disease,^152^ and oral tuberculosis,^151^ also invade the CNS along the peripheral nerve or blood-brain barrier, causing pain, meningitis or intracranial infection. Notably, even oral manipulations, like endodontic treatments, tooth extractions, oral surgery, and simple toothbrushing, may cause acute or chronic infection.^153^ When oral pathogens spread through the blood system or nerve fibers into the brain, severe consequences may occur, such as chronic inflammation, brain abscesses,^150^ ischemic stroke,^154^ neurodegenerative diseases, neuropsychological diseases,^155^ and even mortality. For instance, Porphyromonas gingivalis, a pivotal pathogen in gingivitis and periodontitis, can disrupt the blood-brain barrier via inflammation, which is a characteristic feature of cerebral small vessel disease,^154^ thereby increasing the risk of acute ischemic stroke (Fig. 3a).

#### Perineural invasion resulting from tumors in the oral and maxillofacial regions

Certain types of oral tumors, such as ACC and OSCC, can invade nerves, leading to PNI, which is characterized by tumor cells tracking along nerves and/or enveloping at least one-third of the nerve’s circumference.^156^ Furthermore, ACC is one of the most common salivary gland tumors, particularly in the small salivary glands of the palate and parotid gland. Due to its high propensity for spreading along nerves, ACC is capable of causing PNI in the head and neck region.^157^ Facial nerve invasion caused by ACC leads to facial paralysis, while invasion of the trigeminal nerve causes facial pain. Additionally, invasion of the glossopharyngeal nerve and hypoglossal nerve may result in tongue numbness and tongue movement disorders.^24^

The sixth most common malignant tumor, OSCC, can infiltrate the CNS via the facial and trigeminal nerves, leading to the development of intracranial space-occupying lesions^24^ and leptomeningeal disease.^158^ Although PNI in carcinoma of the lip is rare, malignant cells may trail along the IAN to the brainstem, resulting in leptomeningeal carcinomatosis.^158^ In addition, PNI appears in the advanced stages of tongue cancer.^159^ Patients may feel ear pain, throat pain, and pain in other areas involved in PNI.^160^ Although its mechanisms are not yet understood, PNI has been shown to be linked to an elevated risk of recurrence, regional transfer, distant metastasis, and overall worse prognosis (Fig. 3b).^161^

#### Aberrant stomatognathic system and headache

Headache is a prevalent condition that can be caused by various factors.^162^ Some studies have showed that malocclusion and sleep bruxism may contribute to the development of headache.^163^ Among different types of malocclusion, overbite, posterior crossbite, lingual crossbite, and lower crowding have been identified as potential risk factors for tension-type headaches in children and adolescents.^162,164^ The underlying mechanism may be related to the imbalanced bite, which can lead to tension in the masticatory muscles^165,166^ and subsequently trigger headache.^167,168^ Sleep bruxism, which is characterized by tooth grinding and jaw clenching during sleep,^169^ has also been associated with headache.^163^ This association may be due to the development of trigger points in the head and neck,^170^ which are hyperalgesic zones that can induce headache (Fig. 3c).^171^

#### Sjogren syndrome

Although the abnormal oral and maxillofacial system in SS is not the direct cause of neuropathy, neurological and oral symptoms often coexist in SS.^172^ SS is a chronic inflammatory autoimmune disease characterized by mononuclear lymphocytic infiltration in lacrimal and salivary glands,^172,173^ resulting in dry eyes and dry mouth. As the disease progresses, patients may experience various oral symptoms such as swallow dysfunction, oral malodour, rampant caries, periodontal disease, tongue papilla atrophy, sore tongue, salivary gland swelling or mumps, and poor denture retention.^174^ Additionally, orofacial myofunctional disorders and temporomandibular disorders (TMD) are common among SS patients,^175^ with main symptoms including orofacial pain and mandibular function limitation.^176^ In addition to orofacial regions, the nervous system is affected in SS, with CNS lesions such as aseptic meningitis,^177^ cerebellar syndromes^178^ and neuromyelitis optica and others, as well as peripheral neuropathy including sensory neuropathy, sensorimotor neuropathies, and cranial neuropathies.^172^ SS can even increase the risk of PD, dementia^179^ and depression (Fig. 3d).^180^

### Interaction effects of neurological diseases and craniofacial diseases

**Fig. 4:**
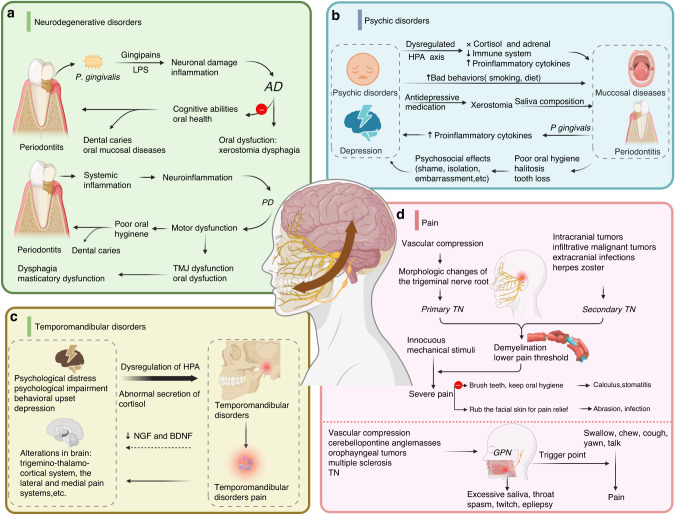
Interaction effects of neurological and craniofacial diseases. **a** The interaction between neurodegenerative disorders and stomatognathic diseases. Periodontitis is conducive to the development of AD and PD, which increase the risk of stomatognathic diseases conversely. **b** Mutual promotion between psychic disorders and stomatognathic diseases. **c** The potential connection between abnormal mental state and temporomandibular disorders. **d** The link between pain and oral symptoms. Vascular compression leads to primary TN. And the etiology of secondary TN is various. No matter what kind of TN, it can cause a series of oral symptoms. *P. gingivalis Porphyromonas gingivalis*, LPS lipopolysaccharide, HPA hypothalamic-pituitary-adrenal. Created with BioRender.com

The pathogeneses of some chronic diseases are exceedingly intricate, making it difficult to identify definitive instigating factors. In fact, in some cases, the diseases may mutually promote each other during their distinct stages. Consequently, this section aims to expound upon the plausible bidirectional associations between these diseases (Fig. 4).

#### Neurodegenerative disorders

AD is the most common neurodegenerative disorder, its clinical characteristic is often manifested as progressive cognitive impairment.^181^ It has been discussed extensively that periodontitis is a risk factor for AD.^182,183^ Bacterial proteins and DNA from periodontal pathogens can provoke neuronal damage and cognitive impairment.^184^ Conversely, the severity of oral diseases is positively linked to AD,^185^ because patients in the advanced stage of AD lose intellectual and social abilities, as well as the ability to maintain proper oral hygiene practices. This leads to oral lesions like caries,^186^ periodontitis,^187^ stomatitis,^188^ ulcerations, angular cheilitis, candidiasis^189,190^ and oral dysfunction.^191^ The second most common neurodegenerative disorder, PD, is characterized by motor dysfunction.^192^ Periodontal inflammatory disease is also linked to the morbidity of PD.^193^ The pathogenic mechanism may involve neuroinflammation, which is a prevalent characteristic of various neurodegenerative disorders.^155^ Due to autonomic dysfunction, muscle stiffness, slowness of movement and tremor, PD patients are prone to developing stomatognathic diseases and motor impairments, like caries, periodontitis,^194^ TMJ dysfunction,^195^and oral dysfunctions (Fig. 4a).^196–198^

#### Psychological disorders

In addition to neurodegenerative diseases, there is mutual promotion between psychic disorders and stomatognathic diseases. Psychological factors, emotional stress, and schizophrenia may induce various oral diseases,^199^ such as oral ulcers, migratory stomatitis, polymorphous erythema, mucoid pemphigus, and chronic periodontitis.^200–202^ Among these psychological factors, the dyadic relation between depression and periodontal disease has been extensively studied.^203^ Depression is a relevant pathogenetic factor for periodontitis,^25^ and in turn, oral diseases can exacerbate the progression of depression (Fig. 4b).

#### Temporomandibular disorders

TMD are associated with an individual’s mental state. In fact, the biopsychosocial model of TMD was proposed long ago to describe how psychological distress,^204^ psychosocial impairment, and behavioral upset are highly prevalent among TMD patients.^205–207^ Stress and negative affect are considered potentially important risk factors for TMD.^208^ But the specific mechanism has not been fully clarified, which may refer to dysregulation of the hypothalamic-pituitary-adrenal^209^ and aberrant secretion of cortisol.^210^ However, the effect of TMD and associated pain on the nervous system is relatively weak. Patients with painful TMD have been found that salivary levels of NGF and brain-derived neurotrophic factor (BDNF) are lower compared to healthy control subjects^211^ NGF^212^ and BDNF^213^ are related to psychological impairment, which reflects a potential connection between an abnormal mental state and TMD. And patients suffering from painful TMD surely experience heightened self-perceived cognitive impairments and depressive symptoms.^214^ Furthermore, extensive alterations in brain structures have been observed in individuals afflicted with TMD pain,^215^ including modifications in the trigemino-thalamo-cortical system, the lateral and medial pain systems, periaqueductal gray-raphe magnus pathway and the motor system. Nevertheless, the relation between these neuropeptides and psychological distress is more complicated than previously thought, and further research is required to understand the intricate interaction between TMD and psychological distress (Fig. 4c).

#### Pain

Oral and maxillofacial pain is a significant issue that perplexes many patients and seriously impacts their facial muscle movement and daily routines. Pain-sensitive structures in the oral and maxillofacial region are distributed in the intracranial trigeminal and glossopharyngeal nerves, and in the extracranial oral and maxillofacial skin, subcutaneous tissue, muscle, TMJ, dental pulp, and oral mucosa.^216^ Therefore, diseases that stimulate pain-sensitive structures may cause oral and maxillofacial pain. The most common facial pain is trigeminal neuralgia (TN), which is divided into primary TN and second TN.^26^ Primary TN is typically caused by vascular compression with morphologic changes of the trigeminal nerve root.^217^ Second TN may be caused by an intracranial tumor,^218^ such as those in the cerebellopontine angle or multiple sclerosis, infiltrative malignant tumors, trauma, and rheumatologic diseases. Even extracranial infections can lead to TN, especially odontogenic infections, such as endodontic infections, and periodontal infections or abscesses.^219^ Acute pulpitis is a distinct form of dental inflammation that can elicit severe and spontaneous sharp pain upon compression of the involved nerve without timely drainage. Patients experience radiating pain along the second or third branch of the trigeminal nerve to the ipsilateral head, ear, face, and temporal region,^220^ often leading to secondary TN. Besides, herpes zoster infection can affect the trigeminal ganglion to trigger secondary TN.^219^ The underlying pathology of both primary TN and secondary TN is widely acceptable to be demyelination,^218^ which triggers impulses with high-frequency afterdischarges.^221,222^ Therefore, innocuous mechanical stimuli in the trigeminal territory, including light touch, cold air, brushing teeth, and eating, can trigger severe pain.^217^ As a result, patients may avoid basic hygiene practices, like washing their face, brushing their teeth, and smiling, leading to poor facial and oral hygiene accompanied by calculus and stomatitis. Furthermore, during the pain attack phase, patients may vigorously rub their facial skin to alleviate the pain, leading to partial abrasion and secondary infection.

Glossopharyngeal neuralgia (GPN) is a relatively rare condition that may be affected by both the nervous system and oral structure. Patients with GPN experience paroxysmal pain in the tonsils, pharynx, tongue base, and other areas. Similar to TN, one of the recognized lesions associated with GPN is nerve compression by a blood vessel at the root entry zone of the brainstem.^223,224^ Furthermore, CPN has also been linked to cerebellopontine angle masses, oropharyngeal tumors, multiple sclerosis, and TN.^225–227^ Also, GPN has trigger points that can elicit pain, such as swallowing, chewing, coughing, yawning, and talking. In addition to neuralgia, other symptoms may occur, such as excessive saliva, throat spasm,^228^ twitch, and epilepsy (Fig. 4d).^229^

## Conclusions and future perspectives

This review summarizes the connection between neurodevelopment and craniofacial development, highlighting the intricate crosstalk between nerves and jawbones, as well as diseases among the two systems. The current research on the association between the nervous system and the stomatognathic system is extensive and intricate; however, it also has limitations. The underlying causes of congenital diseases in the stomatognathic system, such as Moebius syndrome, Parry–Romberg syndrome, and AS, remain unclear. Moreover, the connection between facial deformities and other neurodevelopmental disorders has not been established; this lack of understanding causes more complex disease management and higher costs, particularly without the aid of genetic screening. There is also a scarcity of studies that incorporate pathways related to the immune system and cation channels in jaw-regulating nerves. Research about the regulation of the CNS on the jawbone is also inadequate. At the molecular level, there is a lack of in-depth studies regarding the effect of acetylcholine and SP on the jawbone. In particular, the regulation of SP on the jawbone is perplexing, as opposing effects of SP have been observed at different concentrations. Interestingly, even at the same tested concentration, SP exerts different effects on the regulation of the jawbone. The role of SP may be strongly influenced by the specific surrounding environment, the duration of exposure, and the state of the jawbone. In addition, the interaction of neurological diseases and craniofacial diseases further complicates the issue, and the initial factors and the specific mechanism remain unclear.

Therefore, prioritizing neurodevelopment and neurological diseases related to the stomatognathic system is crucial for the timely prevention and treatment of oral diseases. It is imperative to investigate the contribution of published gene mutations to congenital diseases in both systems and expand the scope of gene mutation research. Such work would enhance the efficiency of prevention and treatment through embryo intervention and prenatal screening for dysplasia, as well as the early detection and diagnosis of refractory diseases, such as AD and TN. In addition, further investigations are necessary to examine the effects of bioactive factors, such as acetylcholine and SP, in regulating jawbone acquisition and loss. Furthermore, neural pathways mediated by the immune system and cation channels within jawbones are worth investigating. This may lead to the development of innovative strategies for neuro-bone tissue engineering.

Moreover, we found that the relationship between these two systems was far more complicated than what has been previously demonstrated. Based on existing research and obvious controversy, it is evident that the interaction mechanism between the nervous system and the stomatognathic system merits further investigation and potentially opens new research avenues.
