# Publisher Correction: The interaction between the nervous system and the stomatognathic system: from development to diseases

**DOI:** 10.1038/s41368-023-00250-3

**Published:** 2023-09-11

**Authors:** Yuzhu Wu, Yanhua Lan, Jiajie Mao, Jiahui Shen, Ting Kang, Zhijian Xie

**Affiliations:** Stomatology Hospital, School of Stomatology, Zhejiang University School of Medicine, Zhejiang Provincial Clinical Research Center for Oral Diseases, Key Laboratory of Oral Biomedical Research of Zhejiang Province, Cancer Center of Zhejiang University, Engineering Research Center of Oral Biomaterials and Devices of Zhejiang Province, Hangzhou, China

**Keywords:** Extracellular signalling molecules, Bone remodelling, Bone quality and biomechanics

Correction to: *International Journal of Oral Science* 10.1038/s41368-023-00241-4, published online 15 August 2023

Following the publication of the original article^1^, the authors reported that some of the in-text citations were incorrect and caused by typesetting mistakes.

The following corrections have been implemented:

On page 5, citations 135–139 in Table 2 should be changed to 106–110.

On page 7, citations 106–110 should be removed, and citation 80 should be replaced by 261.

On page 10, citation 185 should be removed.

The original article^1^ has been updated.
